# Dynamic Microbiome Changes Reveal the Effect of 1-Methylcyclopropene Treatment on Reducing Post-harvest Fruit Decay in “Doyenne du Comice” Pear

**DOI:** 10.3389/fmicb.2021.729014

**Published:** 2021-08-27

**Authors:** Yang Zhang, Congcong Gao, Md. Mahidul Islam Masum, Yudou Cheng, Chuangqi Wei, Yeqing Guan, Junfeng Guan

**Affiliations:** ^1^Plant Genetic Engineering Center of Hebei Province, Institute of Biotechnology and Food Science, Hebei Academy of Agriculture and Forestry Sciences, Shijiazhuang, China; ^2^Department of Plant Pathology, Bangabandhu Sheikh Mujibur Rahman Agricultural University, Gazipur, Bangladesh

**Keywords:** microbiome, 1-MCP, pear, fruit quality, decay

## Abstract

Pathogen-induced decay is one of the most common causes of fruit loss, resulting in substantial economic loss and posing a health risk to humans. As an ethylene action inhibitor, 1-methylcyclopropene (1-MCP) can significantly reduce fruit decay, but its effect on fruit pathogens remains unclear. Herein, the change in microbial community structure was analyzed using the high-throughput sequencing technology, and characteristics related to fruit quality were determined after 1-MCP (1.0 M l L^–1^) treatment in “Doyenne du Comiceis” pear fruit during storage at ambient temperature. Overall, 1-MCP was highly effective in reducing disease incidence and induced multiple changes of the fungal and bacterial microbiota. At day 15, the microbial diversity of fungi or bacteria was reduced significantly in the control fruit (non-treated with 1-MCP), which had the most severe decay incidence. For fungi, in addition to *Alternaria* being the most abundant in both 1-MCP treatment (59.89%) and control (40.18%), the abundances of *Botryosphaeria* (16.75%), *Penicillium* (8.81%), and *Fusarium* (6.47%) increased significantly with the extension of storage time. They became the primary pathogens to cause fruit decay in control, but they were markedly decreased in 1-MCP treatment, resulting in reduced disease incidence. For bacteria, the abundance of *Gluconobacter* (50.89%) increased dramatically at day 15 in the control fruit, showing that it also played a crucial role in fruit decay. In addition, *Botryosphaeria*, *Fusarium* fungi, and *Massilia*, *Kineococcus* bacteria were identified as biomarkers to distinguish 1-MCP treatment and control using Random Forest analysis. The redundancy analysis (RDA) result showed that the amount of *Botryosphaeria*, *Penicillium*, and *Fusarium* were positively correlated with disease incidence and respiration rate of pear fruits while negatively correlated with fruit firmness. This investigation is the first comprehensive analysis of the microbiome response to 1-MCP treatment in post-harvest pear fruit, and reveals the relationship between fruit decay and microbial composition in pear fruit.

## Introduction

As one of the three deciduous fruit trees, pear is an important fruit crop grown throughout the temperate zone. *Pyrus communis* L. cv. “Doyenne du Comice” is very popular among consumers because of its gorgeous appearance, rich fragrance, soft and juicy flesh, high commodity value, and health care value (López et al., 2001). However, the fruit decay caused by pathogenic agents has seriously limited the post-harvest shelf life.

Pathogen-induced decay is the most crucial reason for post-harvest fruit loss among many other factors such as environmental conditions, sprouting, quality loss, and overripening (Sommer, 1985; Buchholz et al., 2018). Disease losses in pears are mainly caused by fungi, including blue mold caused by *Penicillium expansum*, gray mold caused by *Botrytis cinerea*, bitter rot caused by *Glomerella cingulate*, and Mucor rot caused by *Mucor piriformis* (Mari et al., 2003; Sardella et al., 2016; Luciano-Rosario et al., 2020). In recent years, there has been an increasing number of reports of fungal infections in pears on different cultivars worldwide. The interactions between pathogens, including fungi and bacteria, and plants have been extensively studied, but much remains to be explored about the diversity of fruit microbiome during post-harvest storage, especially the pathogens causing fruit decay (Willersinn et al., 2015; Buchholz et al., 2018).

To date, various chemical and physical methods have been used to maintain the quality of fruits and to reduce the damage of pathogens (Wassermann et al., 2019; Aslam et al., 2020). By competing with ethylene for binding receptors, 1-methylcyclopropene (1-MCP), as an ethylene action inhibitor, delays ethylene-mediated physiological and biochemical responses related to fruit ripening. Thus, 1-MCP has been widely used to store and preserve fruits and vegetables as a new fresh-keeping agent (Jiang et al., 2004; Khan and Singh, 2007; Zhang et al., 2012; Li et al., 2013). Several reports have shown that 1-MCP reduces fruit decay in various fruits under proper concentration (Dou et al., 2005; Xu et al., 2017; Min et al., 2018). However, these studies mainly focused on the inhibition effect of 1-MCP on some particular pathogens or diseases; the impact of 1-MCP on the microbial diversity of post-harvest fruits remains unclear. Therefore, we anticipate that this study will contribute to a deeper understanding of 1-MCP using post-harvest fruit storage.

Initially, the effect of fresh-keeping agents on fruit microorganisms was studied using traditional approaches, such as pathogen isolation, colony counts of bacteria and molds, and denaturing gradient gel electrophoresis (DGGE) technology (Sheffield et al., 1989; Feroz et al., 2016; Lin et al., 2018). However, these methods required re-culturing microorganisms in a nutrient medium, which led to the loss of many slow-growing but important microorganisms. The microbiome can greatly delineate the composition, structure, and diversity of microbial populations in various environments. The development of DNA sequencing technology has made the microbiome an efficient and direct means to explore biological diversity in post-harvest fruit (Droby and Wisniewski, 2018; Zhang et al., 2020; Taîbi et al., 2021).

In the present study, we have performed a DNA metabarcoding approach to investigate the fruit microbiome changes induced by 1-MCP treatment in post-harvest pear storage. Furthermore, the relationship among the disease incidence, fruit quality, physiological characteristics, and microbial composition was also demonstrated after the 1-MCP treatment in the pear fruit.

## Materials and Methods

### Material Collection and Treatments

The “Doyenne du Comice” pear (*P. communis* L.) fruits were harvested at maturity stage (July 2020) from an orchard of Shenzhou City (115.490266°E, 38.05122°N), Hebei Province, China, and transported to the Lab directly. Fruits with similar weight (about 120 g per fruit) were randomly divided into two groups: one was treated with 1.0 μl L^–1^ 1-MCP (SmartFresh, AgroFresh, United States) at 25°C for 14 h and another was set as control (CK) without 1-MCP. Then, the fruits were stored at 25 ± 0.5°C and relative humidity of 90 ± 5% for 0, 5, 10, and 15 days. The treatments were marked as CK0d, CK5d, CK10d, and CK15d, representing fruits stored for 0, 5, 10, and 15 days in the control group, while MCP0d, MCP5d, MCP10d, and MCP15d represent fruits stored for 0, 5, 10, and 15 days in 1-MCP treated group, respectively.

### Analysis of Microbial Diversity

#### DNA Extraction and Illumina Sequencing

For sampling, four pieces of fruit tissue, including pulp and peel (about 40 g, 1 cm thick) were symmetrically taken from each fruit, and the samples were homogenized with a blender. Total microbial DNA was extracted from the fruit homogenate. Each treatment contained five replicates, and each replicate had five fruits. The 16S rRNA genes V4–V5 region was amplified with the primers 799F (5′-AAC MGG ATT AGA TAC CCK G-3′) and 1193R (5′-ACG TCA TCC CCA CCT TCC-3′). The ITS1 region of the fungal community was amplified with the primer ITS1F (5′-CTT GGT CAT TTA GAG GAA GTA A-3′) and ITS2 (5′-GCT GCG TTC TTC ATC GAT GC-3′). The PCR products were sequenced on an Illumina MiSeq/NovaSeq platform at Personal Biotechnology, Shanghai, China.

#### Sequence Analysis

After the barcode sequence was removed, sequence denoising was carried out according to the QIIME 2 DADA2 analysis process (Bolyen et al., 2019). The number of amplicon sequence variants (ASVs) at each of the seven taxonomic levels including domain, phylum, class, order, family, genus, and species was counted according to the results of taxonomic annotations, and the flower plot was performed by the genes cloud tools, a free online platform for data analysis^1^. Taxonomic composition was analyzed after all samples were adjusted to the same sequencing depth, and microbial dynamics at each taxonomic level were shown. Biomarker identification was analyzed by using Random Forest with the function of “classify-samples-ncv” in Q2-sample-classifier. Correlations of fungi and bacteria were performed by the gene cloud tools. Relationship among fruit quality, physiological characteristics, and microbial community diversity was analyzed using redundancy analysis (RDA) and plotted by the gene cloud tools.

### Fungi Isolation and Identification

A small piece of fruit tissue was cut off from the diseased spot, soaked in 70% alcohol for 30 s, washed with sterile water three times, and then placed on the PDA medium. After 3–5 days of incubation at 25°C, mycelium was picked from the edge of the colony and transferred to a fresh PDA medium for purification. After two or three times of purification, a single pure colony was obtained as no contamination of other fungi was detected under a microscope (OLYMPUS BX51, Japan). The purified colonies were inoculated in PDA inclined medium for 2 days and then stored at 4°C for further use.

Based on the isolation of four strains of pathogenic fungi, 0.1 g of activated fungal hyphae was taken and ground into powder with liquid nitrogen. Total fungal DNA was extracted using the fungal genomic DNA rapid extraction kit (Sangon Biotech, Shanghai) according to the instructions. DNA samples were amplified with primers of ITS1/ITS4 and sequenced by Sangon Biotech (Shanghai). The obtained sequences were BLAST at NCBI to complete fungal identification.

### Fungal Membrane Integrity Determined by Fluorescence Microscope

Fungal membrane integrity was performed according to the method described by Li and Tian (2006). Fungal spores were treated with 1.0 μl L^–1^ 1-MCP for 14 h, and stained with 10 μg ml^–1^ of propidium iodide (PI) (Sangon Biotech, Shanghai) for 10 min at 30°C in the dark. The spores were observed and photographed using a microscope (OLYMPUS BX51, Japan), equipped with a luciferin rhodonine filter set (OLYMPUS U-RFL-T, Japan). The fungal membrane integrity rate (MIR) was calculated by the following formula: MIR = [1 − (the number of red spores/the number of total spores)] × 100%.

### Disease Incidence

Disease incidence was calculated as the ratio of the number of fruits with visible disease spots to the total number of fruit (*n* = 30). Each treatment had five replicates.

### Assessment of Fruit Quality and Physiological Characteristics

#### Fruit Quality

Fruit quality including firmness, soluble solid content (SSC), and titratable acidity (TA) were measured as our previous study (Cheng et al., 2019). For firmness, pear fruit was peeled about 1-mm thick at the equator and was determined with a handheld firmness meter (GY-4, Tuopu, China). SSC was measured using a PAL-1 handheld digital saccharimeter (ATGAO, Japan). TA was measured using the method of acid–base titration. Each treatment was conducted in triplication, and each replication consists of five fruits.

#### Respiration Rate and Ethylene Production Rate

The respiration rate was analyzed using an infrared CO_2_ analyzer (HWF-1A, Kexi Instruments, China), and the ethylene content was analyzed using a gas chromatograph (GC-9790II, Fuli Instruments, China) and calculated to ethylene production rate. Each treatment had three replicates and 10 fruits per replication.

#### Weight Loss Ratio

The weight loss ratio of pear fruit was calculated by comparing the fruit weight at each time point after treatment with that at day 0. Each treatment had three replicates with 10 fruits per replicate.

### Statistical Analysis

The graphs were generated by using the GraphPad Prism 8 software (GraphPad Inc., CA, United States). The significant differences between treatments were tested by two-way analysis of variance (ANOVA), and differences were considered to be significant at *p* < 0.05 (^∗^), *p* < 0.01 (^∗∗^), and *p* < 0.001 (^∗∗∗^). In addition, sequence data analyses were performed using QIIME 2 and R packages (v4.0.3).

## Results

### 1-Methylcyclopropene Reduced Decay of Pear Fruit

**FIGURE 1:**
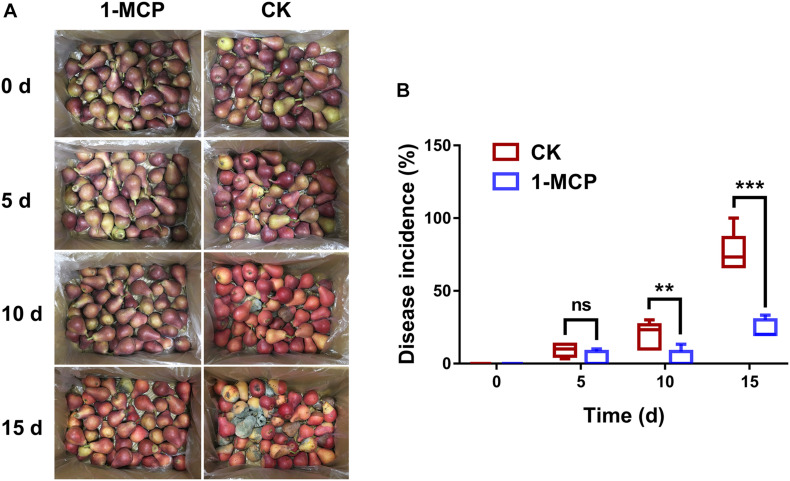
Pear (*Pyrus communis* L.cv. “Doyenne du Comice”) fruit decay was reduced by 1.0 μl L^–1^ 1-methylcyclopropene (MCP) during storage. **(A)** Symptoms of pear decay during storage. **(B)** Disease incidence of pear fruit during storage. The disease incidence rate was calculated using the formula as the number of fruits with visible disease spots/the total number of fruits × 100. Each treatment had five replicates. The asterisk represents the significance between the different treatments (**p < 0.01; ***p < 0.001).

To determine the effect of 1-MCP in resistance to fruit disease, the disease incidence of fruit decay was compared between 1-MCP treatment and CK after 0, 5, 10, and 15 days of storage. As shown in Figure 1A, many hyphae were observed on the surface of the fruits in the CK group, but few in the 1-MCP treatment group after 10 and 15 days of storage, indicating that fruits treated differently showed different resistance to pathogens. Further results showed that there was no significant difference in disease incidence at day 5 between the 1-MCP group and the control group, while there was a significant difference (*p* < 0.01) at day 10 and a very significant difference (*p* < 0.001) at day 15 (Figure 1B).

### 1-Methylcyclopropene Affected Microbial Diversity in Pear Fruit

#### Number of Amplicon Sequence Variants

In order to show the effect of 1-MCP on the microbial diversity in fruit, we compared the number of ASVs with different treatments (Supplementary Table 1). As shown in Supplementary Figure 1, the number of ASVs of both fungi and bacteria in CK at day 15 (CK15d) was much smaller than that in other treatments, indicating that some dominant species of fungi or bacteria affected the microbial diversity in CK at day 15.

#### Taxonomic Composition of Fruit Microorganisms

**FIGURE 2:**
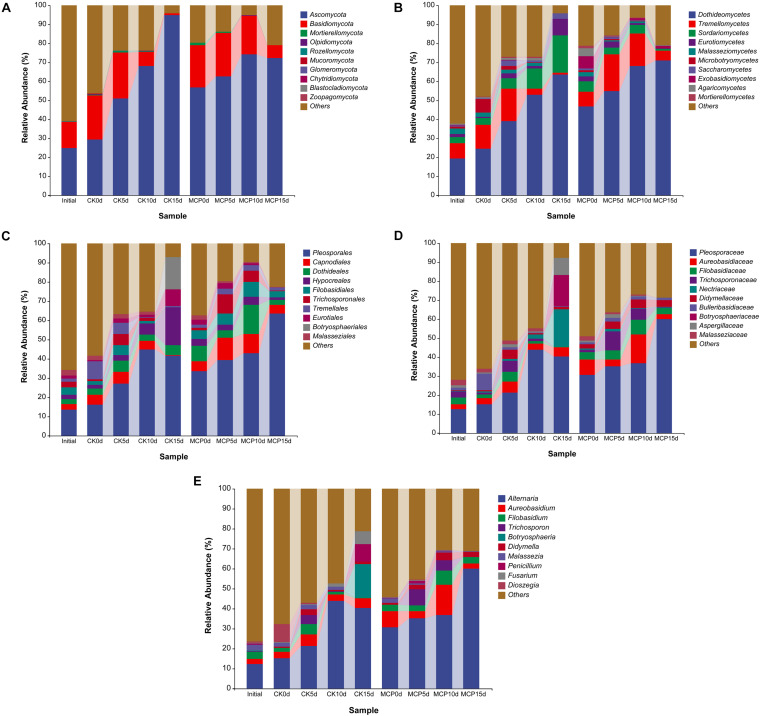
Taxonomic composition of fungi at phylum **(A)**, class **(B)**, order **(C)**, family **(D)**, and genus **(E)** levels. Pear (*Pyrus communis* L. cv. “Doyenne du Comice”) fruits were treated with or without 1.0 μl L^–1^ of 1-MCP during storage. Fruits were stored at 25 ± 0.5°C and relative humidity of 90 ± 5% for 0, 5, 10, and 15 days. The treatments were marked as CK0d, CK5d, CK10d, and CK15d, representing fruit stored for 0, 5, 10, and 15 days in the control group, while MCP0d, MCP5d, MCP10d, and MCP15d represent fruits stored for 0, 5, 10, and 15 days in the 1-MCP-treated group, respectively.

**FIGURE 3:**
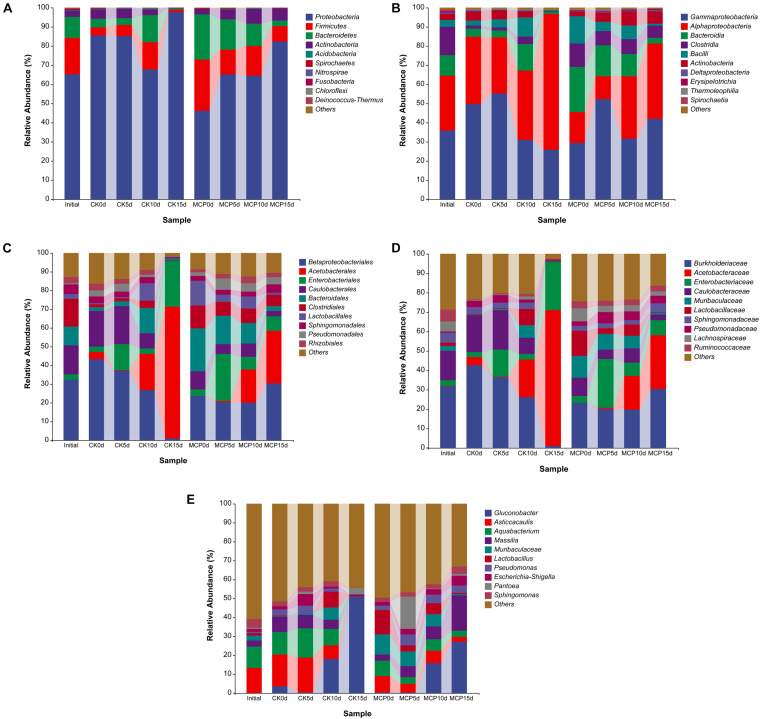
Taxonomic composition of bacteria at phylum **(A)**, class **(B)**, order **(C)**, family **(D)**, and genus **(E)** levels. Pear (*Pyrus communis* L. cv. “Doyenne du Comice”) fruits were treated with or without 1.0 μl L^–1^ of 1-MCP during storage. Fruits were stored at 25 ± 0.5°C and relative humidity of 90 ± 5% for 0, 5, 10, and 15 days. The treatments were marked as CK0d, CK5d, CK10d, and CK15d, representing fruit stored for 0, 5, 10, and 15 days in the control group, while MCP0d, MCP5d, MCP10d, and MCP15d represent fruits stored for 0, 5, 10, and 15 days in the 1-MCP-treated group, respectively.

The dominant microorganisms are much more likely to be the pathogens causing fruit decay. Therefore, the 10 most abundant fungal or bacterial taxa were analyzed at the levels of phylum, class, order, family, and genus (Figures 2, 3). The same sequencing scale was used for subsequent analysis to compare the dynamic changes of fungi or bacteria in different treatments.

For fungi, *Ascomycota* and *Basidiomycota* were superior in all samples at the phylum level. Interestingly, in contrast to the 1-MCP treatment group, the abundance of *Ascomycetes* in the control group increased over time, while the abundance of *Basidiomycetes* decreased, indicating that *Ascomycetes* became the dominant pathogen causing the fruit decay and that 1-MCP had a significant effect on the fungal microbiota in pear fruit. At the class level, *Dothideomycetes* (63.66%), *Sordariomycetes* (19.73%), and *Eurotiomycetes* (8.82%) increased significantly in CK15d, while *Tremellomycetes* decreased over time. *Pleosporales* (41.70%), *Dothideales* (5.07%), *Hypocreales* (19.71%), *Eurotiales* (8.81%), and *Botryosphaeriales* (16.75%) were the most common order in CK15d, while *Pleosporales* (63.63%), *Capnodiales* (4.39%), *Dothideales* (2.69%), and *Filobasidiales* (3.24%) were abundant in MCP15d. At the family level, *Pleosporaceae* (40.18%), *Aureobasidiaceae* (5.07%), *Nectriaceae* (19.68%), *Botryosphaeriaceae* (16.75%), and *Aspergillaceae* (8.81%) were the most abundant fungal group in CK15d, while *Pleosporaceae* (59.89%), *Aureobasidiaceae* (2.68%), *Filobasidiaceae* (3.24%), and *Didymellaceae* (3.58%) were in MCP15d. At the genus level, *Alternaria* (40.18%), *Aureobasidium* (5.05%), *Botryosphaeria* (16.75%), *Penicillium* (8.81%), and *Fusarium* (6.47%) accounted for the largest proportion in CK15d, while *Alternaria* (59.89%), *Aureobasidium* (2.68%), *Filobasidium* (3.07%), and *Didymella* (2.69%) accounted for MCP15d. In particular, *Alternaria*, *Botryosphaeria*, *Penicillium*, and *Fusarium* increased significantly in CK15d compared with CK0d, indicating that these fungi were likely to be the major pathogens that caused fruit decay.

For bacteria, *Proteobacteria* was the overwhelming majority at phylum level across all samples, while *Firmicutes* and *Bacteroidetes* were squeezed out after 15 days of storage. At class level, *Gammaproteobacteria* (25.72%) and *Alphaproteobacteria* (70.92%) occupied the prominent positions in CK15d, while *Gammaproteobacteria* (42.02%), *Alphaproteobacteria* (39.33%), *Clostridia* (6.25%), and *Actinobacteria* (6.38%) remained abundant in MCP15d. At order level, *Acetobacterales* (70.33%) and *Enterobacteriales* (24.84%) squeezed *Betaproteobacteriales* (0.76%) out of competition in CK15d, while *Betaproteobacteriales* (30.32%) remained abundant in MCP15d even though *Acetobacterales* (27.88%) increased considerably. Similarly, the family *Acetobacteraceae* (70.33%) and *Enterobacteriaceae* (24.84%) increased significantly in CK15d, while *Burkholderiaceae* (30.09%), *Acetobacteraceae* (27.87%), and *Enterobacteriaceae* (7.77%) were the most prevalent bacterial groups in MCP15d. In CK15d, *Gluconobacter* (50.89%) was the dominant bacterial taxonomic community at the genus level, whereas in MCP15d, *Gluconobacter* (26.99%) and *Massilia* (18.71%) were the most common, indicating that 1-MCP induced multiple changes in bacterial microbiota in pear fruit.

### Biomarker Identification Associated With 1-Methylcyclopropene-Treated Pear Fruit

**FIGURE 4:**
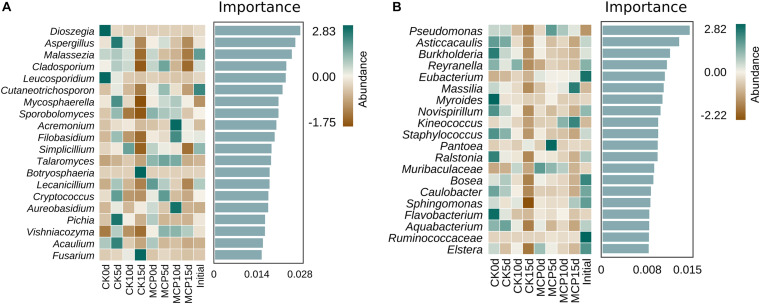
Random forest analysis of fungi **(A)** and bacteria **(B)** in pear (*Pyrus communis* L.cv. “Doyenne du Comice”) fruit after treatment with or without 1-MCP during storage. The horizontal axis of the histogram shows the importance of species to the classifier model, and the vertical axis is the taxon name of fungi or bacteria at the genus level. The heat map shows the abundance distribution of these genera in each group. Five-fold cross-validation was used. Results from the Random Forest analysis showed that *Botryosphaeria* and *Fusarium* were presumably the fungal biomarkers, and *Massilia* and *Kineococcus* were the bacterial biomarkers of CK15d, indicating that these organisms were critical in distinguishing between applications with or without 1-MCP treatment after 15 days of storage.

Random Forest algorithm is a well-known and powerful machine learning algorithm based on the decision tree, and it is particularly well suited to microbial community data, which often presents discrete and discontinuous distributions (Breiman, 2001). In this study, Random Forest was used to analyze the biomarkers of intergroup differences between 1-MCP treatment and control at the genus level. As shown in Figure 4, the top 20 most important genera are listed, and their abundance distribution is plotted as a heat map, which could be considered as the biomarkers of each corresponding treatment. *Botryosphaeria* and *Fusarium* were presumably the biomarkers of CK15d, implying that these two fungi were critical in distinguishing between applications with or without 1-MCP treatment after 15 days of storage. The bacterial genera *Massilia* and *Kineococcus* were the crucial biomarkers to distinguish between CK15d and MCP15d.

### Correlations of Fungi and Bacteria

To explore functional relationships between microorganisms, we analyzed the correlations of the 10 most abundant bacteria and fungi at the genus level (Supplementary Figure 2). The results revealed a positive or negative relationship between specific bacteria and fungi, suggesting that they were functionally synergetic or antagonistic. The bacteria *Aquabacterium* and *Asticcacaulis*, in particular, were strongly positively correlated with a correlation coefficient of 0.86 (*p* < 0.05), while *Lactobacillus* and *Muribaculaceae* came in second with a correlation coefficient of 0.83. There were also positive correlation coefficients between bacteria and fungi such as *Gluconobacter* and *Botryosphaeria* (0.62), *Asticcacaulis* and *Malassezia* (0.50), and *Pseudomonas* and *Didymella* (0.50). On the other hand, we found that *Alternaria* and *Gluconobacter* were found to negatively correlate with other fungi or bacteria, indicating that they were closely related to fruit decay. Indeed, *Alternaria* showed negative correlations with *Malassezia* (−0.52) and *Asticcacaulis* (−0.4), and *Gluconobacter* was negatively correlated with *Aquabacterium* (−0.49), *Asticcacaulis* (−0.47), and *Sphingomonas* (−0.4).

### Effect of 1-Methylcyclopropene on Fungal Membrane Integrity Rate

To determine the effect of 1.0 μl L^–1^ 1-MCP on fungal membrane integrity, the spores with damaged cell membranes were stained with PI and observed using the fluorescence microscope. Sterile water was used as a negative control and carbendazim, a broad spectrum of fungicide, was served as a positive control. The antifungal effect of 1-MCP was confirmed by comparing 1-MCP treatment and the negative control, and the efficiency of the assay was verified by comparing 1-MCP treatment or the negative control to the positive control. After 14 h of incubation under various treatments, the MIR of *Penicillium* spores was 96.3% in the negative control, 90.7% in 1-MCP treatment, and 66.7% in the positive control (Supplementary Figure 3). There was no significant difference of MIR between the negative control and 1-MCP treatment, while there was a significant difference between 1-MCP treatment and the positive control. For *Fusarium*, the MIR was determined as 98.7% in the negative control, 97.3% in 1-MCP treatment, and 83% in the positive control. Similarly, there was no significant difference of MIR between the negative control and 1-MCP treatment, while there was significant difference between 1-MCP treatment and the positive control. For *Alternaria*, the MIR was determined as 70.7% in the negative control, 66.7% in 1-MCP treatment, and 68% in the positive control. No significant difference in MIR was observed among them. These results suggest that 1.0 μl L^–1^ of 1-MCP had little effect on the fungal membrane integrity.

### Relationship Among Fruit Quality, Physiological Characteristics, and Microbial Community Diversity

**FIGURE 5:**
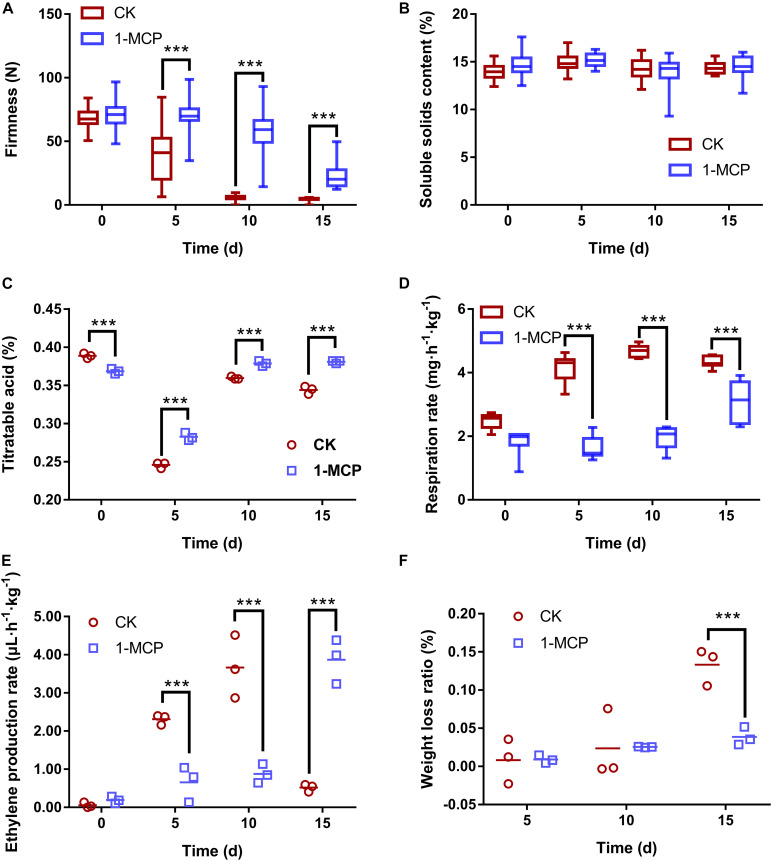
Changes in pear (*Pyrus communis* L.cv. “Doyenne du Comice”) fruit in firmness **(A)**, soluble solids content **(B)**, titratable acidity (TA) **(C)**, respiration rate **(D)**, ethylene production rate **(E)**, and weight loss ratio **(F)** with/without 1-MCP treatment. The asterisk denotes the significance of difference among treatments (***p < 0.001).

Fruit firmness, SSC, TA, respiration rate, ethylene production rate, and weight loss ratio were investigated to reveal the relationship among fruit quality, physiological characteristics, and microbial community diversity. The results showed that the firmness was higher in 1-MCP treated pear than in CK storage (Figure 5A), while SSC had no significant difference (Figure 5B). Similarly, 1-MCP-treated fruit showed significantly higher TA than CK during storage (Figure 5C). The respiration rate of post-harvest pear fruits was inhibited considerably by 1-MCP on days 5, 10, and 15, suggesting that 1-MCP could reduce the nutrient loss of pear fruit (Figure 5D). Simultaneously, 1-MCP greatly reduced ethylene production on days 5 and 10 but bursts on day 15, resulting in a slower fruit ripening (Figure 5E). The ethylene production rate decreased significantly in CK on day 15 due to the excessive decay of the fruit. After 15 days of storage, the weight of the fruit had lost 13.3% in the control group, but just 3.9% in the 1-MCP-treated group (Figure 5F).

**FIGURE 6:**
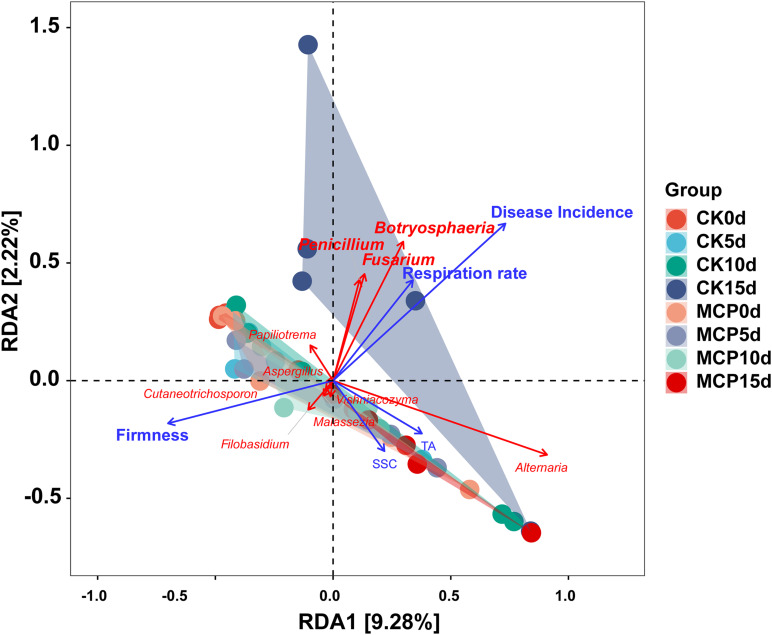
Redundancy analysis (RDA) ordination graph for pear (*Pyrus communis* L.cv. “Doyenne du Comice”) fruit quality, physiological characteristics, and fungal community diversity. Ten most abundant fungal genera (red arrows) and four fruit quality factors (blue arrows) including disease incidence, firmness, soluble solid content (SSC), TA, and respiration rate were plotted. Angles between species and fruit quality factors represent the positive and negative correlation between them (acute angle: positive correlation; obtuse angle: negative correlation; right angles: no correlation). This result indicates the important effect of *Botryosphaeria*, *Penicillium* and *Fusarium* on disease incidence, respiration rate, and firmness during the ripening of fruit.

Redundancy analysis was performed to explore the relationship among disease incidence, fruit quality, physiological characteristics, and microbial community following 1-MCP treatment. As shown in Figure 6, the abundance of three pathogens that cause fruit rot, including *Botryosphaeria*, *Penicillium*, and *Fusarium*, were positively correlated with disease incidence and respiration rate but negatively correlated with firmness.

## Discussion

As a specific inhibitor of ethylene receptor, 1-MCP can bind to ethylene receptor in plant cells, thereby preventing ethylene-dependent responses such as fruit ripening and senescence and, thus, extending shelf-life and maintaining fruit quality (Hassan and Mahfouz, 2010). Several studies have shown that 1-MCP can effectively reduce post-harvest fruit decay. For instance, 1-MCP may help preserve tomato fruit by reducing fruit decay caused by *Alternaria alternata*, *B. cinerea*, and *Fusarium* spp. (Su and Gubler, 2012). In jujubes, 1-MCP effectively suppressed the growth of blue mold-induced fruit rot and significantly reduced the incidence of natural decay (Zhang et al., 2012). To examine the effect of 1-MCP on the decay of d’Anjou pear fruit, the application of 1-MCP was found to reduce bull’s-eye rot, *Phacidiopycnis* rot, stem end gray mold at 300 nl L^–1^, and snow-mold rot at 30 nl L^–1^ (Spotts et al., 2007). Another study found that 1-MCP treatment significantly reduced post-harvest decay of peach fruit and that disease development was reduced when inoculated with *P. expansum* (Liu et al., 2005). In apples, the effect of 1-MCP treatment on post-harvest gray mold and its possible mechanisms were investigated (Zhou et al., 2016). In the current research, 1-MCP substantially reduced the disease incidence of pear (*P. communis* L.cv. “Doyenne du Comice”) by increasing disease resistance, demonstrating that it had a remarkable capacity to mitigate post-harvest diseases of pear fruit.

The next-generation Illumina-based sequencing was used in this study to explore the microbial community of pear fruit treated with or without 1-MCP for 0–15 days of storage. Many fungi and bacteria associated with fruit decay were detected, and their abundance varied among different treatments (Figures 2, 3). In our research, we found the two most common phyla, *Ascomycota* and *Basidiomycota*, in fungi associated with pear fruit samples, both of which are known to cause post-harvest fruit diseases (Ren et al., 2019b; Wassermann et al., 2019; Zambounis et al., 2020). The class *Dothideomycetes* was the most abundant class in both treatments and its abundance increased with the storage time in the present research, which is in line with the studies of endophytic mycobiota in “Jingbai” pear and microbial communities associated with apple and blackcurrant fruits (Vepštaitë-Monstavičë et al., 2018; Ren et al., 2019a). Several families in the *Pleosporales* order of fungi have infected living plants and caused serious plant diseases (Zhang et al., 2009; Wang et al., 2020). *Pleosporaceae* is the largest family in *Pleosporales*, which was characterized as the most abundant family in this study. Members of this family have been reported as plant parasites or saprobes occurring on plant leaves, stems, or fruits (Ariyawansa et al., 2015). At genus level, fungi such as *Alternaria*, *Botryosphaeria*, *Penicillium*, and *Fusarium* were found to grow significantly during storage in this study, which have been detected in a variety of diseased fruits in previous studies (Campenhout et al., 2017; Parveen et al., 2018; Zhao X. et al., 2019; Chen et al., 2020). In comparison with 1-MCP-treated group, *Botryosphaeria* and *Penicillium* were found highly abundant in the control group after 15 days of storage, indicating that 1-MCP may alleviate the disease caused by *Botryosphaeria* and *Penicillium*. The growth patterns of most dominant microorganisms indicated that their abundance increased with the extension of storage time. The main reason for this growth pattern is that the decay-causing pathogens constantly proliferated in the fruit.

Moreover, a high number of *Proteobacteria* were found in many orchard samples including fruits, bark, leaves, and rootstocks by metagenomics (Martins et al., 2013; Liu et al., 2018; Wassermann et al., 2019; Jo et al., 2020). *Proteobacteria* are usually classified into five groups based on rRNA sequences, which are denoted by the Greek letters α, β, γ, δ, and ε. In the present study, two classes of proteobacteria such as *Alphaproteobacteria* and *Gammaproteobacteria* were found to be more prevalent across all the samples (Figure 4), which is consistent with recent studies indicating that bacteria in this taxon are closely related to plant disease (Zhang et al., 2018; Wang et al., 2019). In the current study, the abundance of family *Acetobacteraceae* increased significantly both in control and 1-MCP treatment, indicating that the members in this family played an important role in fruit decay. Further results showed that the *Gluconobacter* genus, an important member of the family *Acetobacteraceae*, increased significantly after 15 days of storage. In accordance with the present results, previous studies have demonstrated that genus *Gluconobacter* were closely related to the fruit decay in apple, pear, and apricot (Van Keer et al., 1981; Liu et al., 2020). However, Bevardi, Frece (Bevardi et al., 2013) reported the biocontrol abilities of *Gluconobacter oxydans* strain isolated from the apple surface against post-harvest infecting fungus *P. expansum*. Current research indicates that members of this genus may grow on the surface of pears as an antagonistic or saprophytic bacterium, but more isolation and identification studies are required to validate this hypothesis.

Biomarker discovery is one of the most important means being used for reflecting the lifestyle and disease of various hosts (Di Paola et al., 2011; Segata et al., 2011; Zhimo et al., 2021). It has been proven that Random Forest can effectively, stably, and accurately classify microbial community samples (Yatsunenko et al., 2012; Chen et al., 2021). *Botryosphaeria* and *Fusarium* were identified as biomarkers in this study to distinguish between 1-MCP treatment and untreated control pear fruits. Notably, members of these two fungal genera have been reported as the pathogens that cause fruit decay in a variety of fruits (Rittenburg, 1983; Garibaldi et al., 2012; Sever et al., 2012; Tang et al., 2012; Zhao X. et al., 2019; Zhao Z.Y. et al., 2019).

Furthermore, correlation matrix revealed a few positive and negative correlation patterns among the dominant fungal and bacterial genera. *Lactobacillus* and *Muribaculaceae* were found highly correlated in the microbial community of pear, which seemed to be consistent with other research that found *Lactobacillus plantarum* restored the gut microbiota imbalance by manipulating relative abundances of certain bacteria in *Muribaculaceae* (Gao et al., 2021). *Gluconobacter* and *Botryosphaeria* were also found to be correlated during the process of fruit decay, indicating that these pathogens could coexist and may be functionally related (Tournas and Katsoudas, 2012). In addition, there were several negative correlation patterns among the microbes, for instance, *Alternaria* and *Malassezia*, *Alternaria* and *Asticcacaulis*, *Gluconobacter* and *Aquabacterium*, *Gluconobacter* and *Asticcacaulis*, and *Gluconobacter* and *Sphingomonas*, which may be due to the high abundance of *Alternaria* and *Gluconobacter* inhibiting the growth of other microorganisms.

In order to show the effect of 1-MCP on fungi, fluorescence staining experiment was conducted to determine the membrane damage of spores caused by 1-MCP. The results showed that 1.0 μl L^–1^ of 1-MCP had little effect on the integrity of fungal cell membrane, suggesting that 1-MCP does not inhibit the development of fungi by damaging cell integrity of spores. Several studies have shown that 1-MCP decreased fruit firmness during storage but was delayed (Villarreal et al., 2010; Moises et al., 2017; Cheng et al., 2019), which has also been confirmed by the present findings (Figure 5). Fruit softening is closely related to the activities of cell wall hydrolases, such as polygalacturonase (PG), pectin methyl esterase (PME), and β-galactosidase (β-Gal). It has been reported that 1-MCP significantly lessened the fruit decay by reducing the activities of PG, PME, and β-Gal (Zhang et al., 2019). Therefore, 1-MCP is likely to minimize fruit decay by inactivating the pectinase activity in fruit, maintaining high fruit hardness, and preventing pathogen infection, rather than inhibiting pathogens directly. The RDA findings in this study obviously revealed the relationship between fruit quality and microbial community after 1-MCP treatment. *Botryosphaeria*, *Penicillium*, and *Fusarium* were shown to be positively correlated with disease incidence and respiration rate, and negatively correlated with fruit firmness (Figure 6), suggesting that these fungal genera are involved in fruit decay and quality change during storage.

## Conclusion

Findings in this study suggest that 1-MCP treatment effectively reduced the disease incidence and induced multiple changes in fungal and bacterial microbiota in “Doyenne du Comice” pear fruit. In contrast to the control, decay-causing fungi such as *Botryosphaeria*, *Penicillium*, and *Fusarium* were suppressed by 1-MCP treatment and their quantity was positively correlated with TA and respiration rate but negatively correlated with fruit firmness. Thus, this study investigated the microbiome responses to 1-MCP during storage and revealed a relationship between fruit decay and microbial composition in “Doyenne du Comice” pear fruit.

## Data Availability Statement

The datasets generated for this study can be found in the Sequence Read Archive (Accession: PRJNA739857).

## Author Contributions

YZ and CG performed the experiments and analyzed the data. JG designed the study and provided the funding. YZ, MM, and JG wrote the manuscript. YC, CW, and YG did the visualization and reviewed the manuscript. All authors read and approved the final manuscript.

## Conflict of Interest

The authors declare that the research was conducted in the absence of any commercial or financial relationships that could be construed as a potential conflict of interest.

## Publisher’s Note

All claims expressed in this article are solely those of the authors and do not necessarily represent those of their affiliated organizations, or those of the publisher, the editors and the reviewers. Any product that may be evaluated in this article, or claim that may be made by its manufacturer, is not guaranteed or endorsed by the publisher.

## Footnotes

1
https://www.genescloud.cn
