# Supplementary material for: Dynamic Microbiome Changes Reveal the Effect of 1-Methylcyclopropene Treatment on Reducing Post-harvest Fruit Decay in “Doyenne du Comice” Pear
Source: Front Microbiol. 2021 Aug 27;12:729014. doi: 10.3389/fmicb.2021.729014 (PMC8430257; doi:10.3389/fmicb.2021.729014)
Supplement: Supplementary file 1 [file Data_Sheet_1.DOCX]

Supplementary Material

# Supplementary Data

Supplementary Material should be uploaded separately on submission. Please include any supplementary data, figures and/or tables. All supplementary files are deposited to FigShare for permanent storage and receive a DOI.

Supplementary material is not typeset so please ensure that all information is clearly presented, the appropriate caption is included in the file and not in the manuscript, and that the style conforms to the rest of the article. To avoid discrepancies between the published article and the supplementary material, please do not add the title, author list, affiliations or correspondence in the supplementary files.

# Supplementary Figures and Tables

## Supplementary Figures


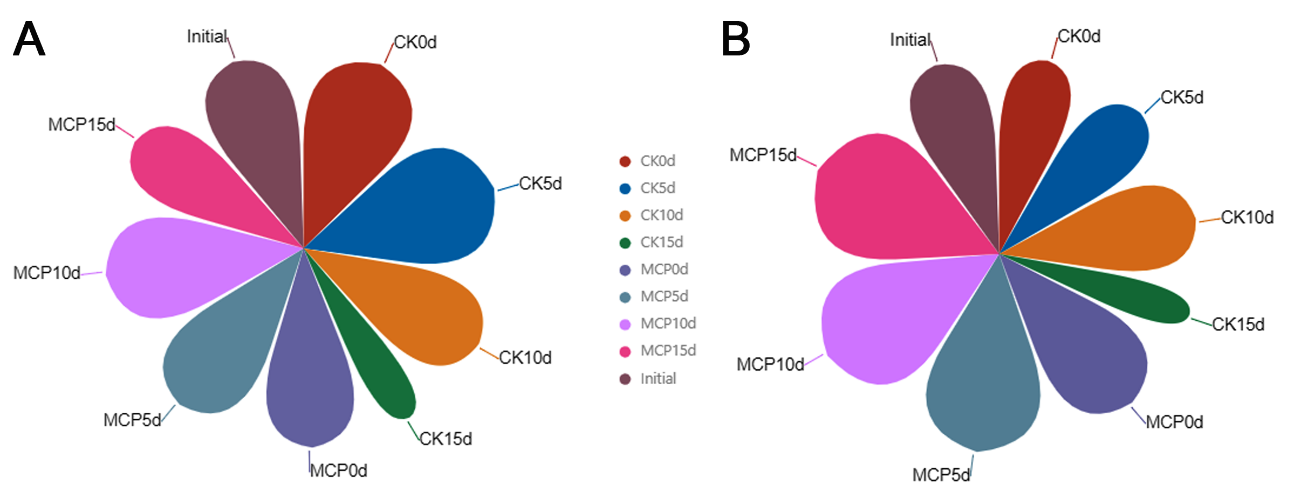


**Supplementary Figure 1.** The number of ASVs of fungi **(A)** and bacteria **(B)** in Pear (*Pyrus communis* L.cv. ‘Doyenne du Comice’) fruit after treated with or without 1-MCP during storage. The treatments were marked as CK0d, CK5d, CK10d and CK15d, which indicated 0, 5, 10 and 15 days after storage in control, and as MCP0d, MCP5d, MCP10d and MCP15d, representing 0, 5, 10 and 10 days after storage in 1-MCP treatment, respectively.


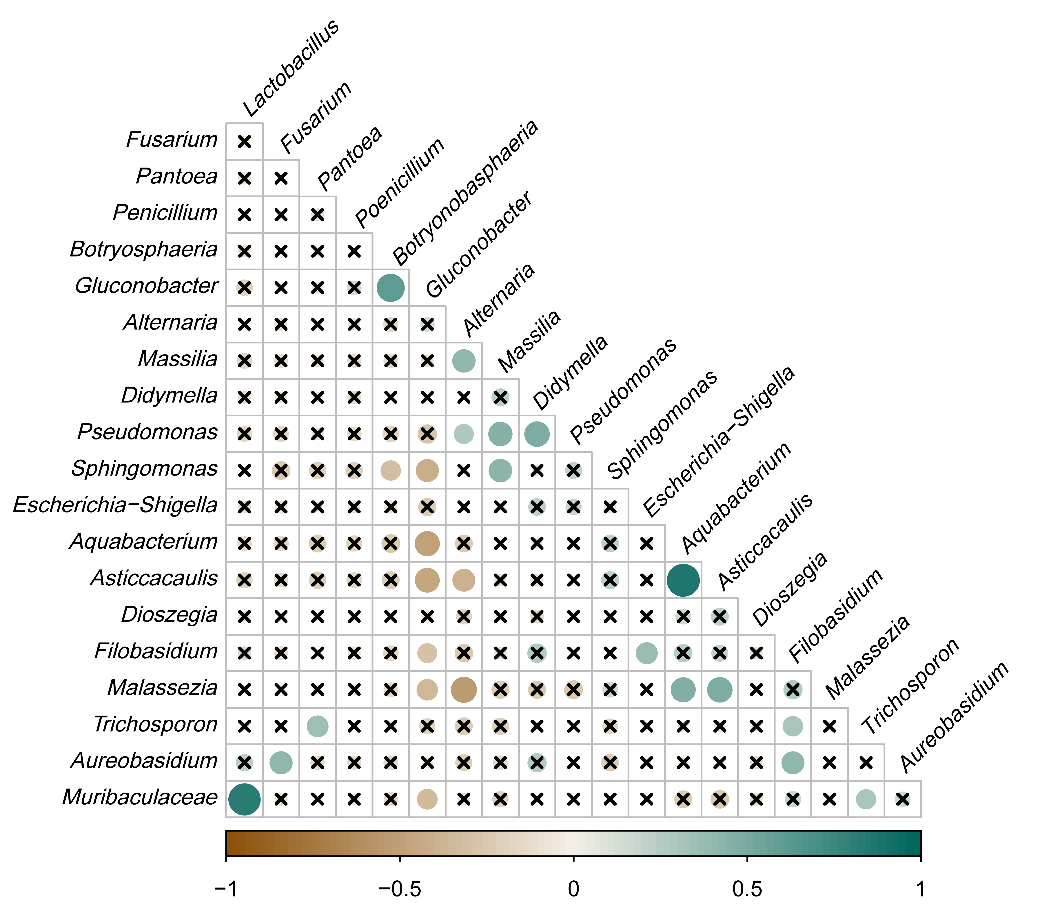


**Supplementary Figure 2.** Correlation matrix heat map of fungi and bacteria at the genus level in Pear (*Pyrus communis* L.cv. ‘Doyenne du Comice’) fruit after treated with or without 1-MCP during storage. Pearson correlation coefficient was analyzed while the significance level was set at 0.05. Values without significant differences are indicated by ×. The heatmap shows the positive values in green, negative in brown. Values range from -1 to 1, where -1 represents a completely negative linear relationship between variables, 1 represents a completely positive linear relationship between variables, and 0 represents no relationship between variables under study.


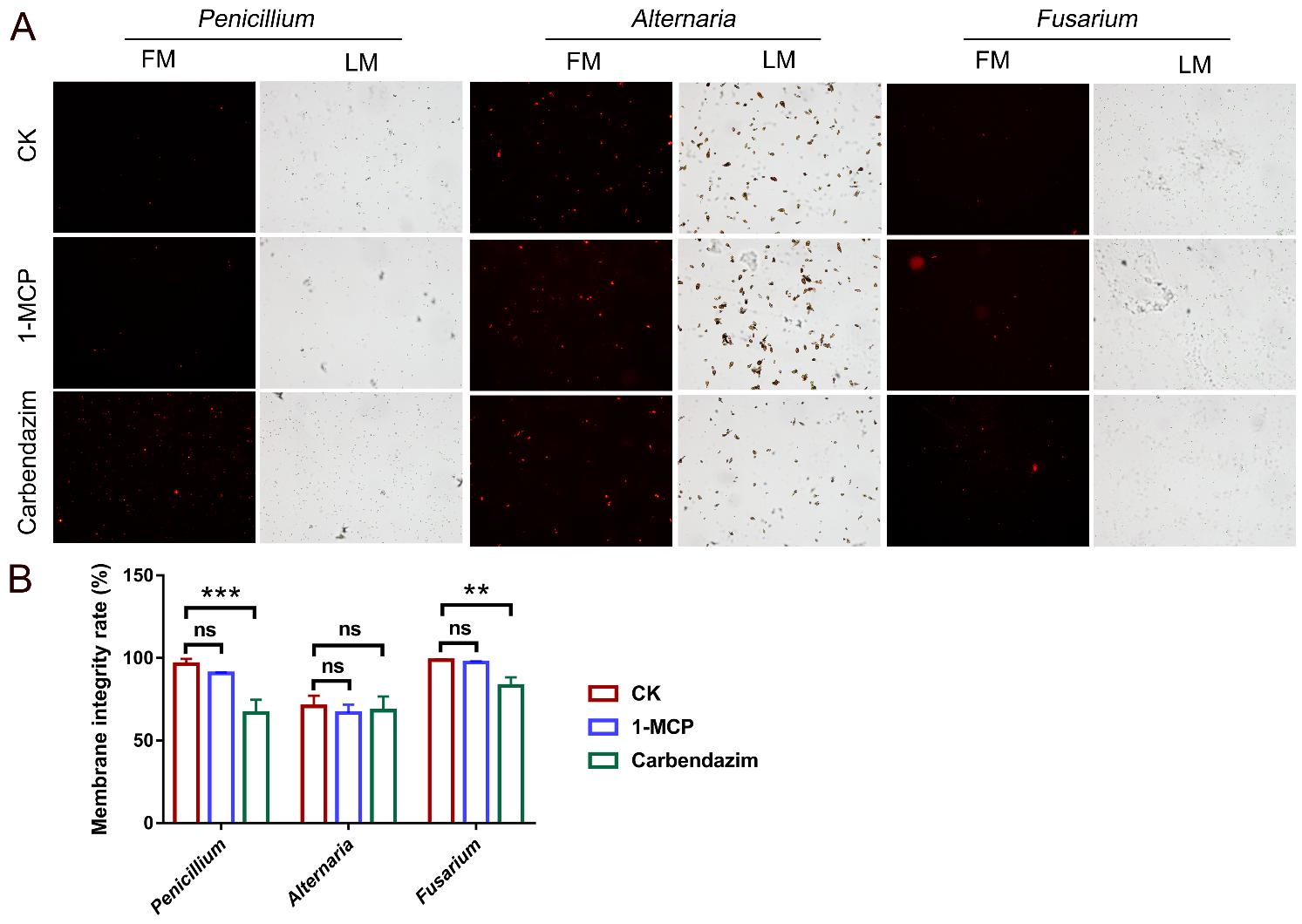


**Supplementary Figure 3.** Effect of 1-MCP on fungal membrane integrity, (A) is the microscopic examination of spores in *Penicillium*, *Alternaria* and *Fusarium* under fluorescence (FM) and light (LM) conditions, (B) is the membrane integrity rate calculated by using the following formula: MIR = [1 − (the number of red spores / the number of total spores)] × 100%. The asterisk denotes the significance of difference among treatments (ns, no significant difference, **, P<0.01, ***, P<0.001).
